# Intra-articular injection of bone marrow aspirate concentrate (BMAC) or adipose-derived stem cells (ADSCs) for knee osteoarthritis: a prospective comparative clinical trial

**DOI:** 10.1186/s13018-023-03841-2

**Published:** 2023-05-11

**Authors:** Andrea Pintore, Donato Notarfrancesco, Arnaldo Zara, Antonio Oliviero, Filippo Migliorini, Francesco Oliva, Nicola Maffulli

**Affiliations:** 1 Department of Medicine, Surgery and Dentistry, University of Salerno, Via S. Allende, 84081 Baronissi, SA Italy; 2 Casa di Cura Salus, 84091 Battipaglia, SA Italy; 3 Department of Orthopaedic, Trauma, and Reconstructive Surgery, University Clinic Aachen, RWTH Aachen University Clinic, Pauwelsstraße 30, 52074 Aachen, Germany; 4 Department of Orthopaedic and Trauma Surgery, Eifelklinik St. Brigida, 52152 Simmerath, Germany; 5 Faculty of Medicine, School of Pharmacy and Bioengineering, Keele University, Thornburrow Drive, Stoke on Trent, England, UK; 6 Centre for Sports and Exercise Medicine, Barts and the London School of Medicine and Dentistry, Mile End Hospital, Queen Mary University of London, 275 Bancroft Road, London, E1 4DG England, UK

**Keywords:** Knee, Osteoarthritis, Mesenchymal stem cells, Bone marrow, Adipose

## Abstract

**Background:**

We determined whether autologous mesenchymal stem cells (MSCs) injections provide clinical and functional improvements in knee osteoarthritis (KOA) patients, and whether the results differ between autologous bone marrow cells (BMAC) and adipose-derived stromal cells (ADSCs).

**Methods:**

Between January 2021 and April 2022, 51 patients undergoing intra-articular injection of BMAC and 51 patients undergoing intra-articular injection of ADSCs were prospectively recruited. The Kellgren and Lawrence (K–L) classification was used to grade the severity of osteoarthritis. Knee Injury and Osteoarthritis Outcome Score (KOOS), Oxford Knee Score (OKS), and visual analog scale (VAS) were collected for all 102 patients in the previous week before the procedures, and at the one and 6 months from injection.

**Results:**

Knee KOOS scores, knee OKS scores, and VAS pain scores changed in similar ways in the two treatment groups. Both treatment groups demonstrated significant improvement pre-procedure to post-procedure in knee KOOS scores (*p* < 0.0001), knee OKS scores (*p* < 0.0001), and VAS pain scores (*p* < 0.0001). Patients with K–L grade 2 showed better functional and clinical outcomes than patients with K–L grades 3 and 4 (*p* < 0.0001).

**Conclusion:**

Both intra-articular BMAC and ADSC injections significantly improved pain and functional outcomes at 6-month follow-up in patients with KOA. The difference between BMAC and ADCSs groups as tissue sources of MSCs was not statistically significant in terms of clinical and functional outcomes.

## Introduction

In osteoarthritis, degenerative joint disease results from breakdown of joint cartilage and underlying bone [1]. Among the over 60, about 10% of males and 18% of females are affected [2, 3], and osteoarthritis causes significant disability [4, 5]. Conventional conservative treatments which include non-steroidal anti-inflammatory drugs, glucosamine, chondroitin sulphate, omega-3 fatty acids, hyaluronic acid, and corticosteroid injections, showed limited clinical benefits [6–8], without preventing the progression of knee osteoarthritis (KOA) or providing long-term improvements in function and joint pain [9]. When KOA progresses to the final stages and non-surgical treatments fail, total knee replacement may be an effective alternative [10, 11]. However, total knee replacement is not without complications, with 20% of patients presenting with persistent pain or loss of function at 12 months [12–14]. With the recent increase in interest for regenerative medicine, patients often undergo intra-articular orthobiologic therapy for KOA and cartilage disease [15, 16]. Mesenchymal stem cells (MSCs) obtained from autologous bone marrow cells (BMAC) or from adipose- derived stromal cells (ADSCs) included in stromal vascular fraction (SVF) [17–20], and platelet-rich plasma (PRP) obtained from autologous blood are used for these purpose [21]. MSCs are multipotent cells that show strong self-renewal capabilities, with a differentiation ability to form chondrocytes, adipocytes, and osteocytes [22]. MSCs may differentiate and participate in the regeneration of connective tissues, given their capability to home in on and attach to diseased tissue [23–25], including bone, articular cartilage, tendon, ligament and fat [22, 26–29]. Furthermore, BMAC and ADSCs exest anti-inflammatory, angiogenic, trophic, and immunomodulatory effects which can retard the progression of OA [30–32]. Though MSCs have been used in clinical practice since 1995 [33], to date there are no real guidelines and indications, and the clinical evidence of MSCs for KOA remains unclear. MSCs could be a safe and efficacious modality for cartilage repair and for regeneration in KOA [34, 35], with improvements in pain and function at short-term follow-up [36–38]. Others authors reported that MSCs for KOA have no clinical evidence [39, 40], and did not recommend their use [41]. To our knowledge, only one study directly compared the results between autologous BMAC or ADSCs as tissue sources of MSCs for symptomatic KOA [42]; there were significant improvements in clinical outcomes with both BMAC and ADSCs injections, without a significant difference in improvement between the two autologous tissue sources. Therefore, we determined whether autologous MSCs injections provide clinical and functional improvements in KOA patients, and whether the results differ between BMAC and ADSCs.

## Materials and methods

### Study design

Between January 2021 and April 2022, 51 patients undergoing intra-articular injection of BMAC and 51 patients undergoing intra-articular injection of ADSCs were prospectively recruited. The present study followed the principles of express in the Declaration of Helsinki and received ethic approval by the Ethic Committee of the University of Salerno (n.90578 del 19/12/2020). All patients signed written consent to participate to the study. The patients’ age, sex, body mass index (BMI), previous surgery on the affected knee, and medical comorbidities were recorded at pre-operative assessment. Selection criteria were clinical findings of KOA with radiographic evidence of degenerative joint disease on standing anteroposterior and lateral radiographies. Exclusion criteria were age over 75 years, Kellgren-Lawrence grade 0 and 1, body mass index < 18 or ≥ 35, patients with a severe (> 10°) varus or valgus deformity, concomitant involvement of ligaments and/or menisci, and infectious or inflammatory joint disease. Patients who received both knee arthroscopy and BMAC or ADSCs at the same time were excluded. The Kellgren and Lawrence (K–L) classification was used to grade the severity of osteoarthritis: grade 0 is absence of radiographic signs of osteoarthritis; grade 1 is characterized by doubtful joint space narrowing and possible osteophytic lipping; in grade 2, there are definite osteophytes and possible narrowing of joint space; grade 3 is defined by moderate multiple osteophytes, definite narrowing of joint space and some sclerosis and possible deformity of bone ends; grade 4 is a severe condition with large osteophytes, marked joint space narrowing, severe sclerosis, and definite deformity of bone ends [43]. Knee Injury and Osteoarthritis Outcome Score (KOOS), Oxford Knee Score (OKS), and visual analog scale (VAS) were collected by one orthopedic surgeon (AP) for 102 patients in the previous week before the procedures, and at the first and 6 months from injection. The KOOS evaluates the course of knee injury and treatment outcomes [44]. It assesses 42 items in 5 separately scored subscales: Pain (nine items), Symptoms (seven items), Activities of daily living (17 items), Sport and Recreation Function (five items) and Quality of Life (four items). Scores are transformed to a 0–100 scale, with zero representing extreme knee problems and 100 representing no knee problems. The OKS is a 12-item patient-reported PRO specifically developed to assess function and pain after knee surgery [45]. The score ranges from 0 (poorest function) to 48 (maximal function). VAS is used to classify knee pain, and it ranges from no pain (0) to an extreme amount of pain (10). Informed consent was obtained from all individual participants included in the study. Before surgery, patient signed an informed consent that informed about the operative procedure, functional and cosmetic expectations, and possible complications related to the surgery, consenting also to be part of any outcome research.

### Adipose- derived stromal cells procedure

**Fig. 1:**
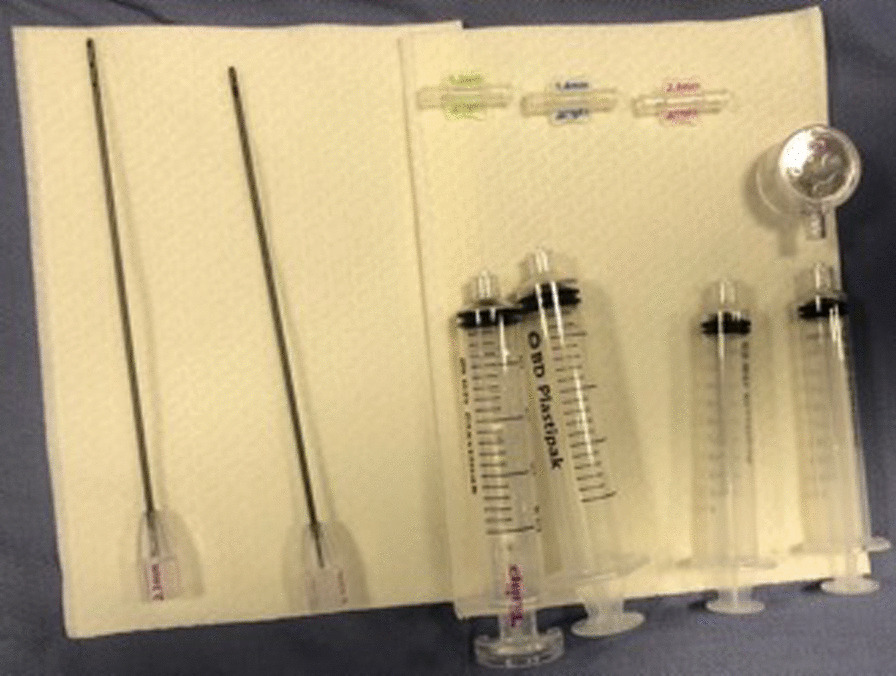
Tulip Soft Harvest GOLD Kit (Tulip Medical)

Patients were placed supine; the abdomen was prepared in a standard fashion with betadine and chlorhexidine. The surgical field was prepared, and 5 ml of Lidocaine 2% was injected at the site of skin incision. All procedures were performed by two fully trained surgeons (DN and AZ) using the Tulip Soft Harvest GOLD System (Tulip Medical) (Fig. 1).

**Fig. 2:**
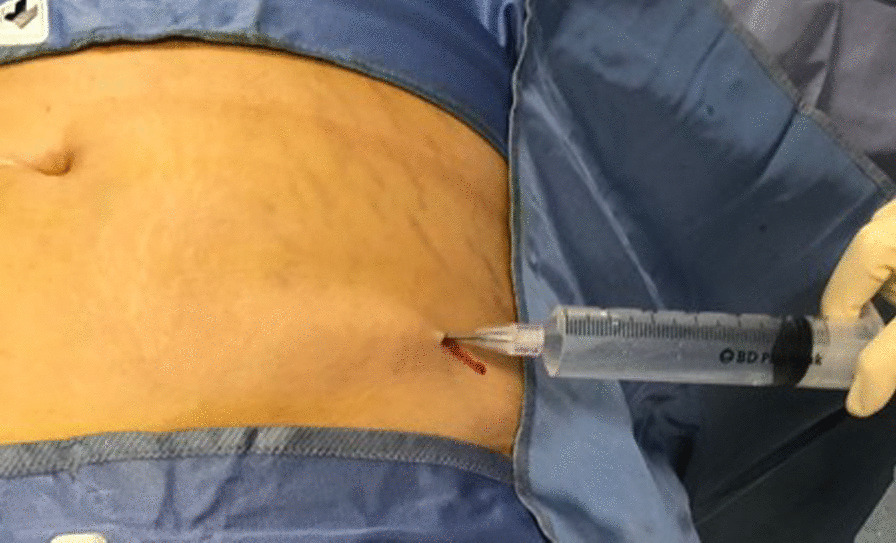
Inject anaesthetic solution

The harvesting area is the abdominal subcutaneous adipose tissue. After performing a small skin incision, a harvesting cannula connected to a 60 ml syringe was used to inject homogenously (Fig. 2). A solution of 250 ml of 0.9% NaCl, 20 ml of 2% Lidocaine, and 0.5 ml of 1 mg/ml Epinephrine.

**Fig. 3:**
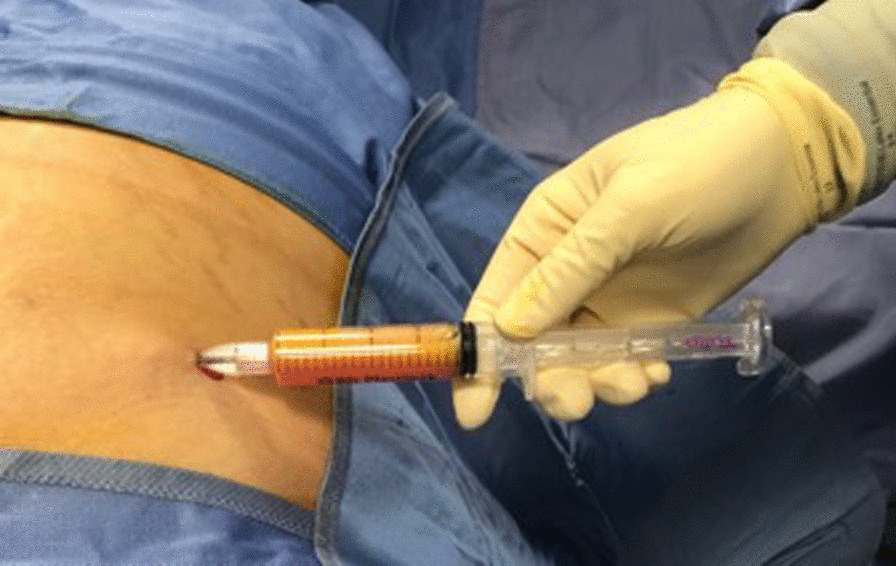
Lipoaspiration

The distribution of 60 ml of the solution in the subcutaneous layers is facilitated by digital manipulation of the abdomen. After 5 min, a harvesting cannula connected to a self-blocking 20 ml syringe is introduced in the subcutaneous fat, and lipoaspiration can start. The block system produces negative pressure inside the syringe, allowing to harvest the lipoaspirate from the previously infiltrated areas (Fig. 3).

**Fig. 4:**
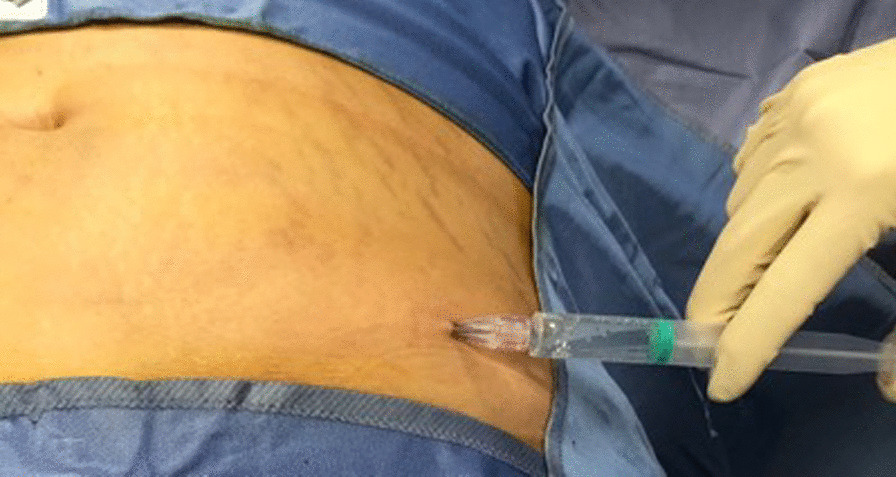
Tranexamic acid injection

After obtaining the lipoaspirate, 10 ml of tranexamic acid is injected (Fig. 4), and a compressive dressing is applied. Fluids harvested were expelled from lipoaspirate by manual pressure, and dry lipoaspirate was collected into 20 ml syringes.

**Fig. 5:**
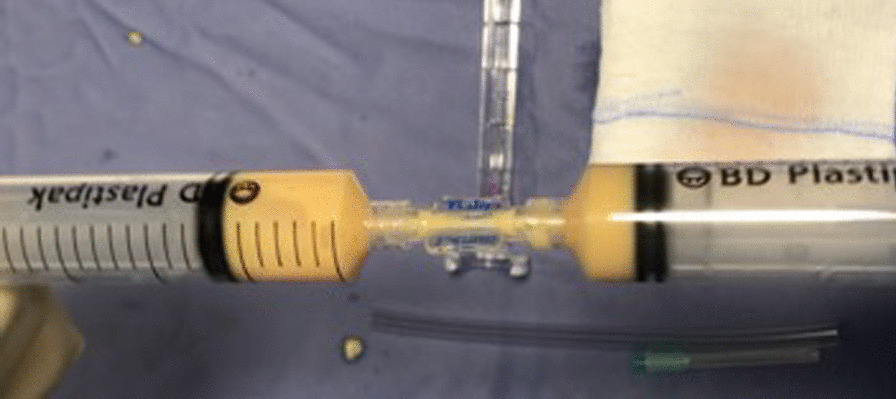
Filtration through transfer devices

The fat-containing syringe was sequentially passed 30 times through a 2.4 mm, 1.4 mm, and 1.2 mm Luer-to-Luer transfer device connected to another empty 20 ml syringe (Fig. 5).

**Fig. 6:**
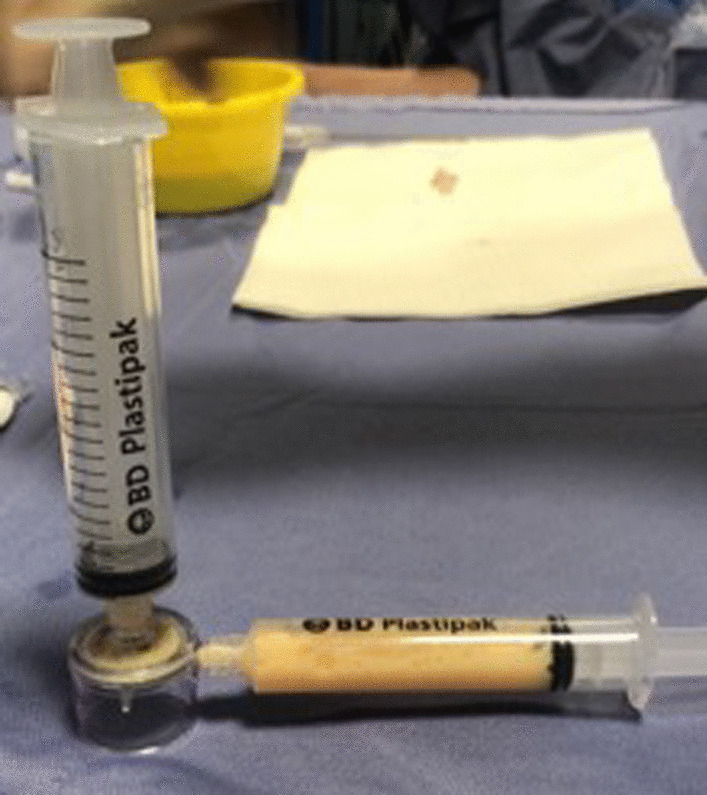
NanoTransfer device

Pre-emulsified lipoaspirate was then collected in a sterile NanoTransfer device and transferred by a single pass through a 0.6- to 0.4 mm mesh screen into an empty 10 ml syringe, ready for injection (10 ml) intra-articularly (Fig. 6).

**Fig. 7:**
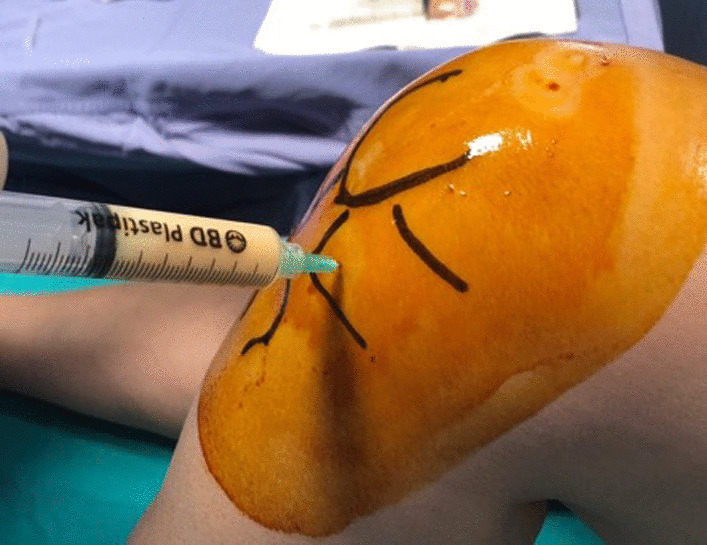
Anterolateral injection

The choice of injection portal may be either superolateral under the patella with the knee extended or through the inferomedial or inferolateral soft part of the knee with the knee flexed to 90° (Fig. 7).

After the injection, the knee is flexed and extended to diffuse the product in the joint. At discharge, all patients were instructed to wear an elastic dressing for a week to reduce the occurrence of hematoma on the abdomen. Patients are partial weight bearing with crutches, and full unaided weight bearing on the treated knee is allowed after 1 week. All patients were instructed to perform isometric quadriceps exercises, and started physiotherapy after 1 week.

### BMAC procedure

**Fig. 8:**
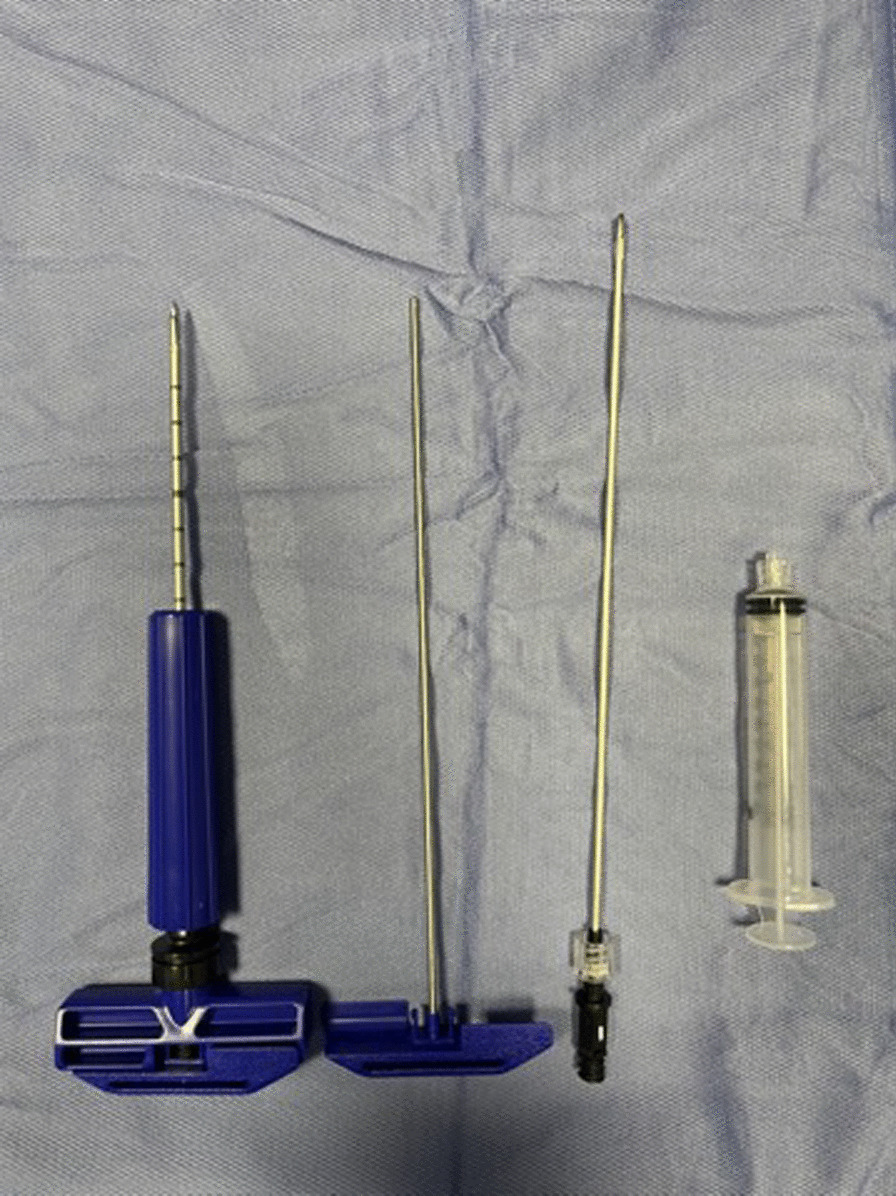
Marrow Cellution™ Bone Marrow Aspiration System

Patients were placed supine under sedation, the surgical field was prepared in a standard fashion with betadine and chlorhexidine. All procedures were performed by two fully trained surgeons (DN and AZ) using the Marrow Cellution™ Bone Marrow Aspiration System (Fig. 8).

**Fig. 9:**
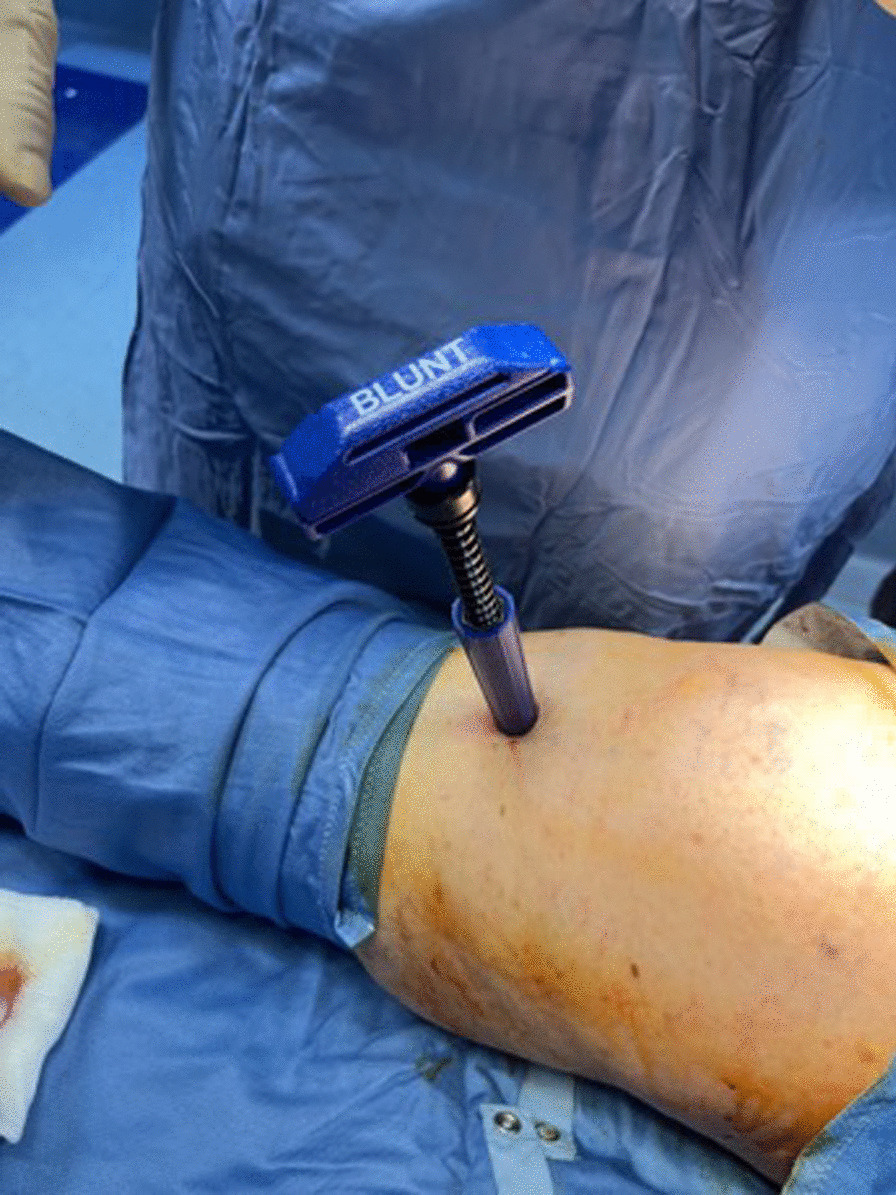
The blunt stylet

The harvesting area is the proximal tibial metaphysis. After performing a small skin incision, a heparin coated needle 13G was introduced just past the cortex into medullary space. The sharp stylet was removed. Then a blunt stylet was inserted, and the access needle was advanced to desired depth, rotating a guide grip to skin level (Fig. 9).

**Fig. 10:**
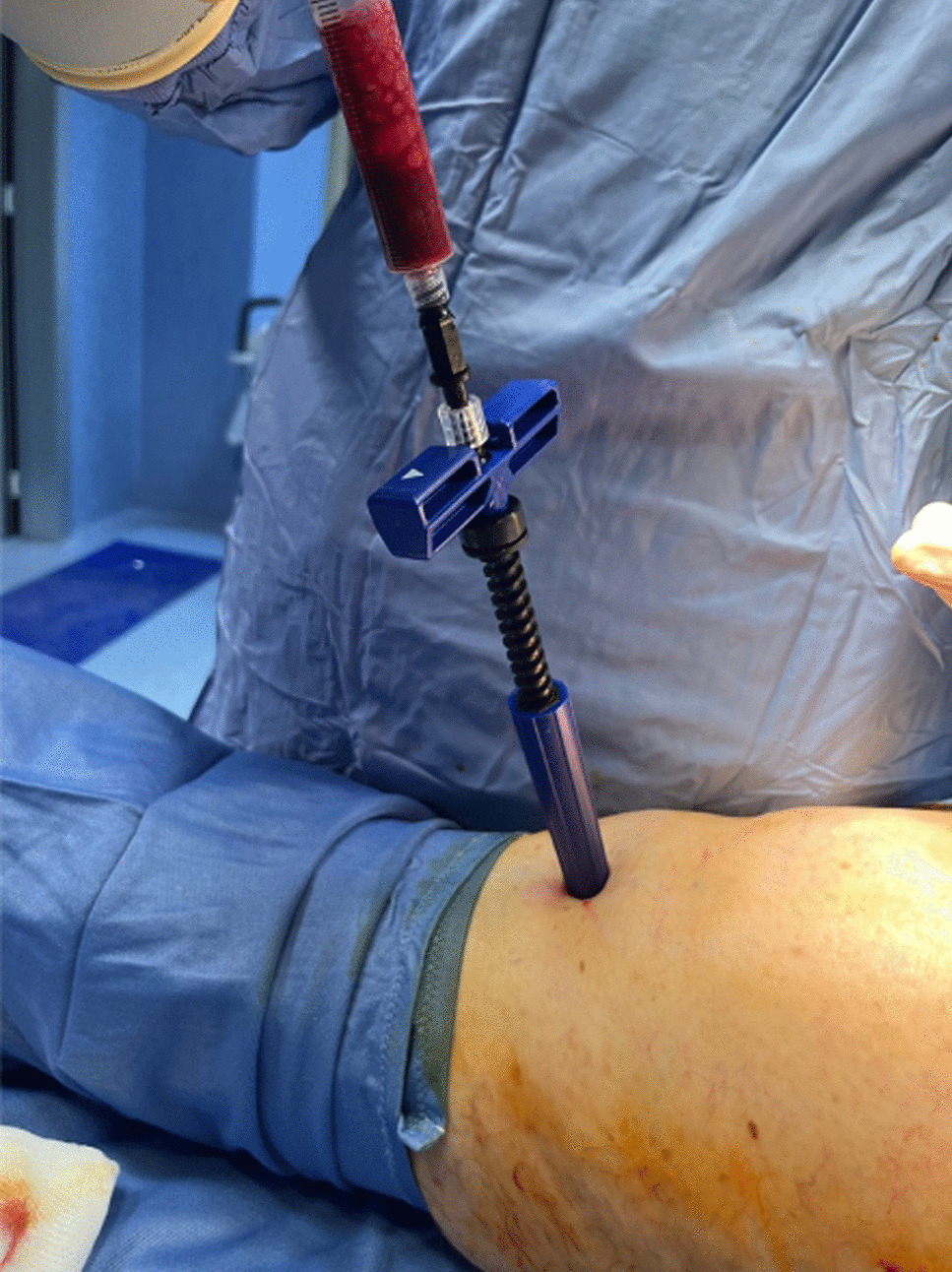
BMAC aspiration

The blunt stylet was removed, and an aspiration cannula connected to a 10 ml syringe. 10 ml of BMAC was aspirated holding a guide grip and rotating the handle 360° counterclockwise gradually to allow the fenestrated stylet to be raised to a new level of undisturbed marrow (Fig. 10).

**Fig. 11:**
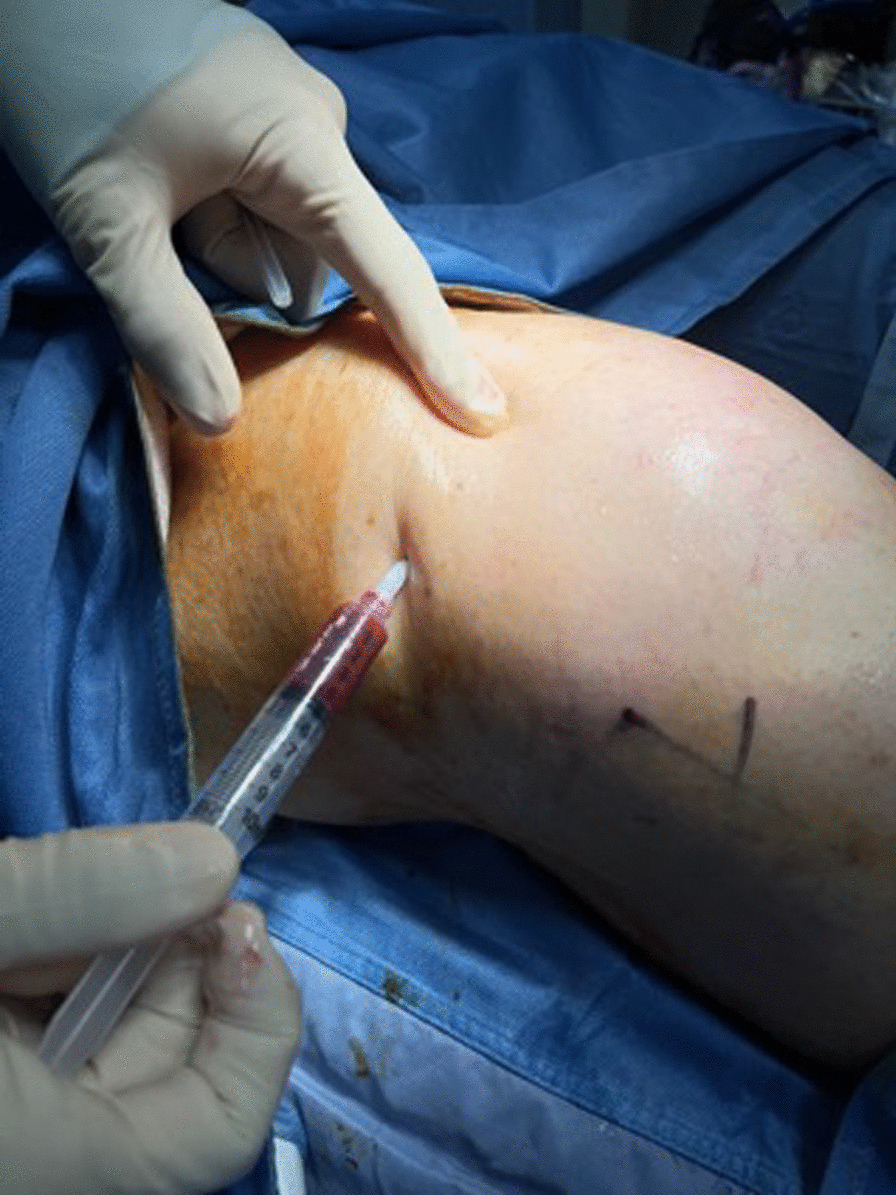
Superolateral injection

The harvest at this point is ready for injection (10 ml) intra-articularly (Fig. 11). The choice of injection portal may be either superolateral under the patella with the knee extended or through the inferomedial or inferolateral soft part of the knee with the knee flexed to 90°.

After the injection, the knee is flexed and extended to diffuse the product in the joint. At discharge, patients are partial weight bearing with crutches, and full unaided weight bearing on the treated knee is allowed after 1 week. All patients were instructed to perform isometric quadriceps exercises, and started physiotherapy after 1 week.

### Statistical analysis

The Student *t* test was used to compare the means of KOOS, OKS, and VAS values before and after surgery. Statistical significance was set at *p* < 0.05.

## Results

### Patient enrollment

**Fig. 12:**
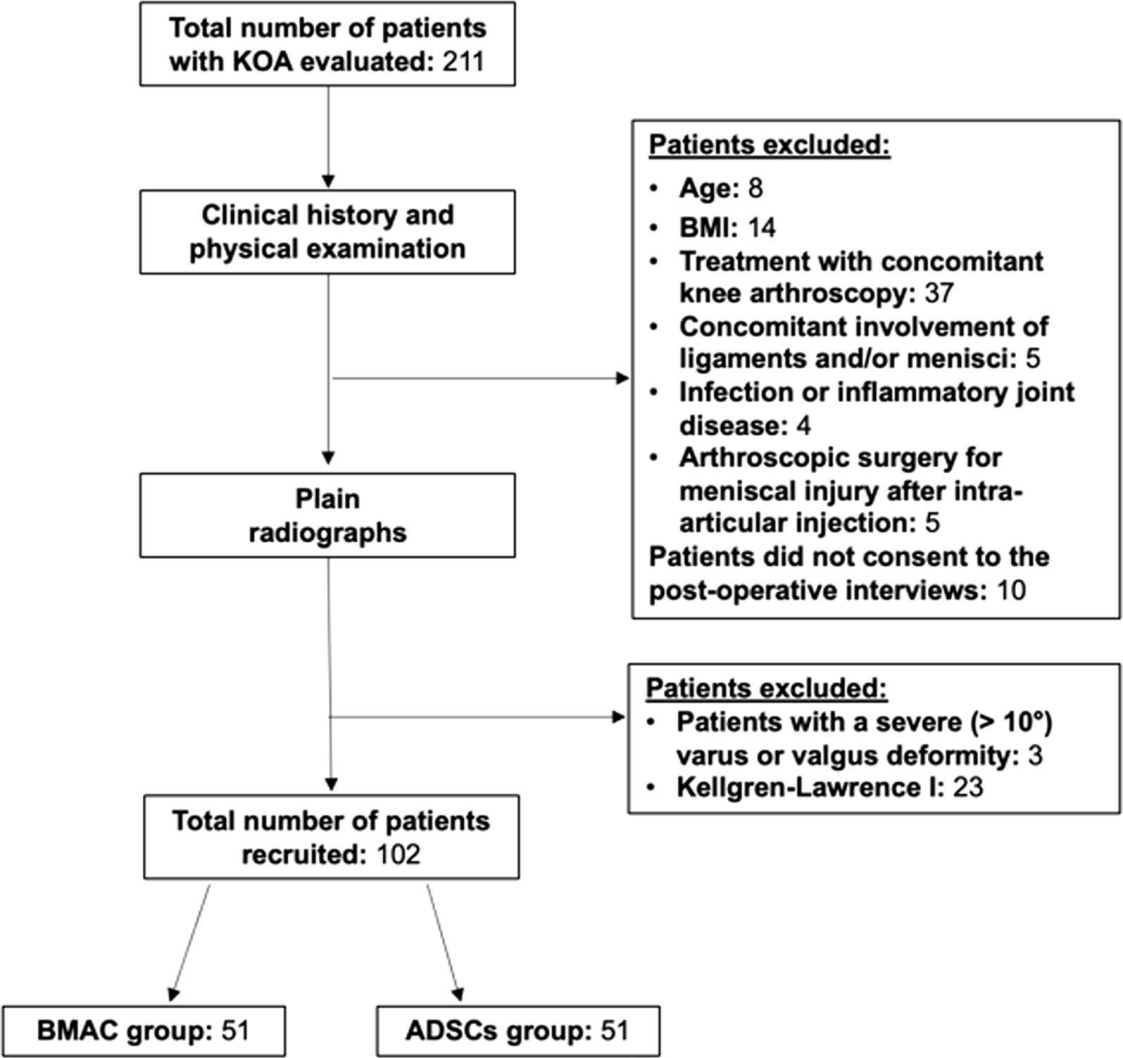
STROBE flow chart

Between January 2021 and April 2022, 211 patients underwent intra-articular injection of ADSCs and BMAC in our department. Of those, 37 patients were excluded because they received both knee arthroscopy and BMAC or ADSCs, and 23 patients were excluded because they were classified as Kellgren-Lawrence grade 1. Eight patients were excluded because older than 75 years, and 14 patients because they had body mass index < 18 or ≥ 35. Nine patients presented concomitant involvement of ligaments and/or menisci, and infectious or inflammatory joint disease. Three patients with a severe (> 10°) varus or valgus deformity were excluded. Ten patients did not consent to the post-operative interviews, and five patients underwent arthroscopic surgery for meniscal injury after intra-articular injection. The remaining 102 patients were included in this study (Fig. 12).

### Patient demographic

Patients were allocated into either of the two treatment groups according to their week of treatment: all patients operated in one week received BMAC injection treatment, all patients operated in the following week received ADSCs injection treatment.

The BMAC group included 51 patients, 22 males (43.2%) and 29 females (56.8%), with a mean age of 57.64 years (range 40 to 68 years). The mean BMI was 28.76. The mean K–L was 2.74. The ADSCs group included 51 patients, 24 males (47.1%) and 27 females (52.9%), with a mean age of 61.94 years (range 50 to 73 years). The mean BMI was 26.76. The mean K–L was 2.55. Patient demographic at baseline is shown in Table 1. We observed no complications during the follow-up.

**Table 1:** Patient demographic at baseline

	BMAC	ADSCs
Number of patients (*N*)	51	51
Number of females (%)	29 (56.8)	27 (52.9)
Mean age (years)	57.64	61.94
Mean BMI	28.76	26.76
Mean K–L system	2.74	2.55

*BMI* Body mass index, *K–L* Kellgren and Lawrence

### KOOS

**Fig. 13:**
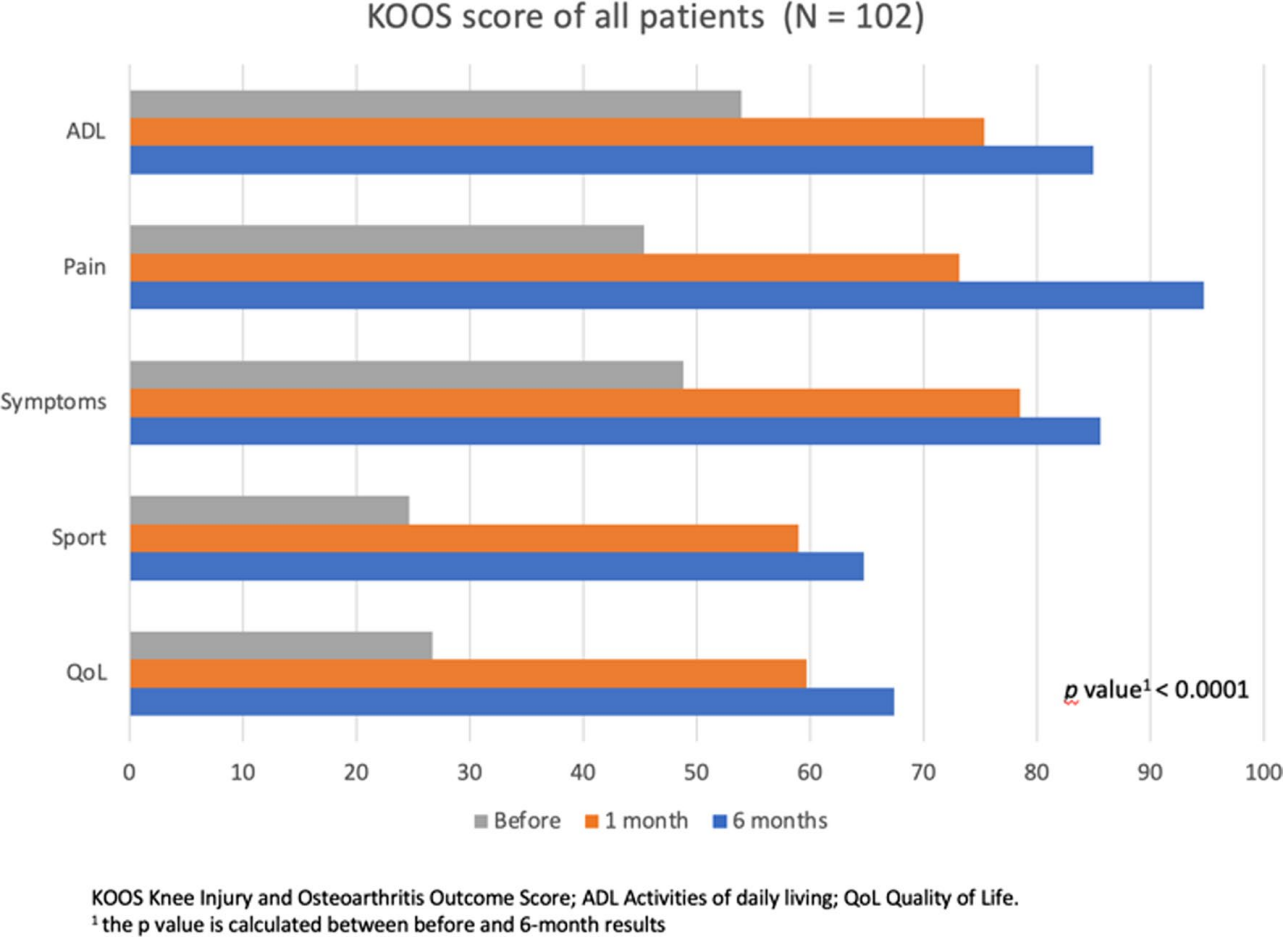
KOOS

The Knee KOOS scores in the two treatment groups improved in similar ways (Fig. 13). The mean KOOS pain of all patients before injection was 45.37 ± 13.47, at the first month from injection it was 73.17 ± 13.52, and at the sixth month from injection it was 94.76 ± 7.89 (*p* < 0.0001). Both treatment groups demonstrated significant improvement from pre- to post-procedure in KOOS pain scores (*p* < 0.0001). The mean KOOS activities of daily living (ADL) scores of all patients before injection was 53.98 ± 15.47, at the first month from injection it was 75.36 ± 12.23, and at the sixth month from injection it was 85 ± 17.51 (*p* < 0.0001). Both treatment groups demonstrated significant improvement from pre- to post-procedure in KOOS activities of daily living (ADL) scores (*p* < 0.0001). The mean KOOS other Symptoms scores of all patients before injection was 48.87 ± 13.91, at the first month from injection it was 78.54 ± 13.94, and at the sixth month from injection it was 85.55 ± 19.88 (*p* < 0.0001). Both treatment groups demonstrated significant improvement from pre- to post-procedure in KOOS other Symptoms scores (*p* < 0.0001). The mean KOOS Function in Sport and Recreation (Sport/Rec) of all patients before injection was 24.70 ± 17.08, at the first month from injection it was 59.01 ± 16.10, and at the sixth month from injection it was 64.7 ± 28 (*p* < 0.0001). Both treatment groups demonstrated significant improvement from pre- to post-procedure in KOOS Function in Sport and Recreation (Sport/Rec) (*p* < 0.0001). The mean KOOS knee-related Quality of Life (QOL) of all patients before injection was 26.74 ± 10.23, at the first month from injection it was 59.71 ± 16.41, and at the sixth month from injection it was 67.40 ± 23.52 (*p* < 0.0001). Both treatment groups demonstrated significant improvement from pre- to post-procedure in KOOS pain scores KOOS knee-related Quality of Life (QOL) (*p* < 0.0001). The difference in Knee KOOS scores between BMAC and ADCSs groups at last follow-up is not statistically significant (Table 2).

**Table 2:** KOOS

	BMAC (*N* = 51)	ADSCs (*N* = 51)	BMAC versus ADSCs (*p* value^c^)
KOOS symptoms^a^			
Before	55.23 ± 15.94	52.73 ± 15.04	0.4167
1 month	77.47 ± 14	73.24 ± 9.8	0.081
6 months	84.13 ± 18.35	85.91 ± 16.77	0.6095
*p* value^b^	< 0.0001	< 0.0001	
KOOS pain^a^			
Before	46.52 ± 17.73	44.23 ± 7	0.3929
1 month	73.07 ± 16.34	73.27 ± 10.12	0.94
6 months	93.60 ± 9.23	95.91 ± 6.16	0.1405
*p* value^b^	< 0.0001	< 0.0001	
KOOS functional^a^			
Before	47.14 ± 17	50.6 ± 9.62	0.2099
1 month	76.97 ± 15.15	80.1 ± 12.56	0.2599
6 months	81.87 ± 22.68	89.24 ± 16	0.0606
*p* value^b^	< 0.0001	< 0.0001	
KOOS sport^a^			
Before	27.25 ± 21.19	22.15 ± 11.28	0.13
1 month	59.50 ± 19.65	58.52 ± 11.71	0.7602
6 months	68.82 ± 35.42	60.58 ± 17.16	0.1383
*p* value^b^	< 0.0001	< 0.0001	
KOOS QoL^a^			
Before	30.83 ± 12.52	22.62 ± 4.51	0.0001
1 month	60 ± 20.94	59.34 ± 10.28	0.8166
6 months	69.36 ± 26.87	65.44 ± 19.7	0.4027
*p* value^b^	< 0.0001	< 0.0001	

*KOOS* Knee Injury and Osteoarthritis Outcome Score, *QoL* Quality of Life

^a^Data was expressed by mean ± standard deviation

^b^*p* value is calculated between before and 6-month results

^c^The *p* value is calculated between the BMAC group results and the ADSCs group results

### VAS

**Fig. 14:**
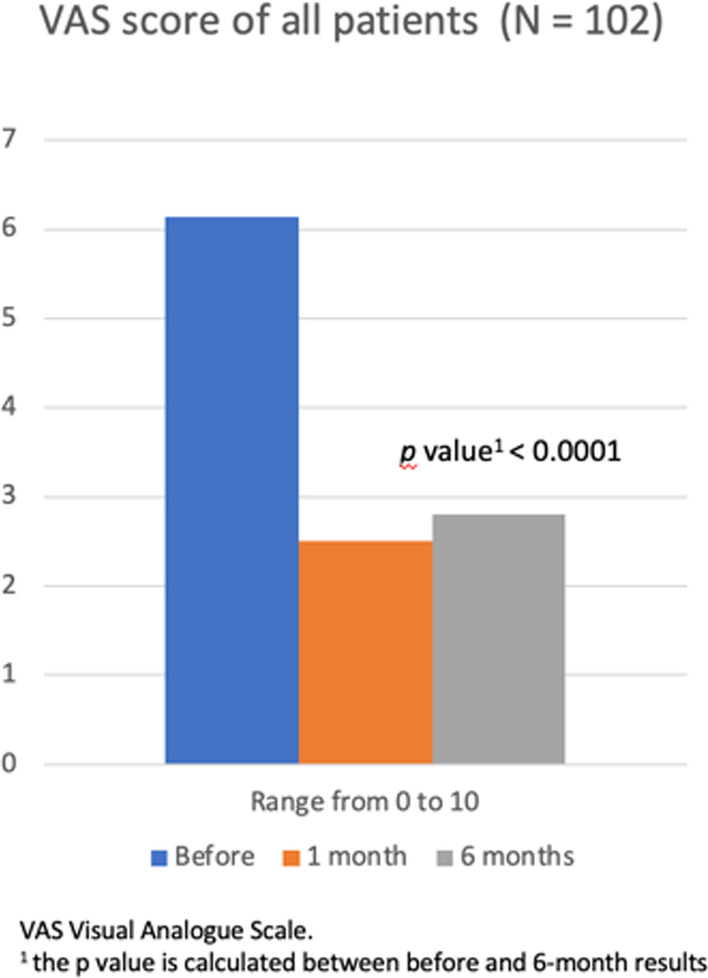
VAS

VAS pain scores in the two treatment groups improved in similar ways (Fig. 14). The overall decrease during follow-up in VAS pain scores was significant. The mean VAS pain score of all patients was 6.14 ± 1.76 points at pre-procedure and 2.8 ± 1.85 at last follow-up (*p* < 0.0001). The difference in VAS pain scores between BMAC and ADCSs groups at last follow-up was not statistically significant (Table 3). Both treatment groups demonstrated significant improvement from pre- to post-procedure in VAS pain scores (*p* < 0.0001).

**Table 3:** VAS and OKS

	BMAC (N = 51)	ADSCs (N = 51)	BMAC versus ADSCs (*p* value^c^)
VAS score^a^			
Before	6.17 ± 1.87	6.1 ± 1.66	0.82
1 month	3.15 ± 0.78	1.84 ± 1	< 0.0001
6 months	2.98 ± 1.97	2.63 ± 1.72	0.34
*p* value^b^	< 0.0001	< 0.0001	
OKS score^a^			
Before	20.53 ± 5.55	20.43 ± 4.92	0.92
1 month	32.88 ± 5.94	34.58 ± 4.38	0.1
6 months	37.57 ± 10	33.35 ± 10.81	0.04
*p* value^b^	< 0.0001	< 0.0001	

*VAS* Visual Analogical Scale, *OKS* Oxford Knee Score

^a^Data was expressed by mean ± standard deviation

^b^*p* value is calculated between before and 6-month results

^c^The *p* value is calculated between the BMAC group results and the ADSCs group results

### OKS

**Fig. 15:**
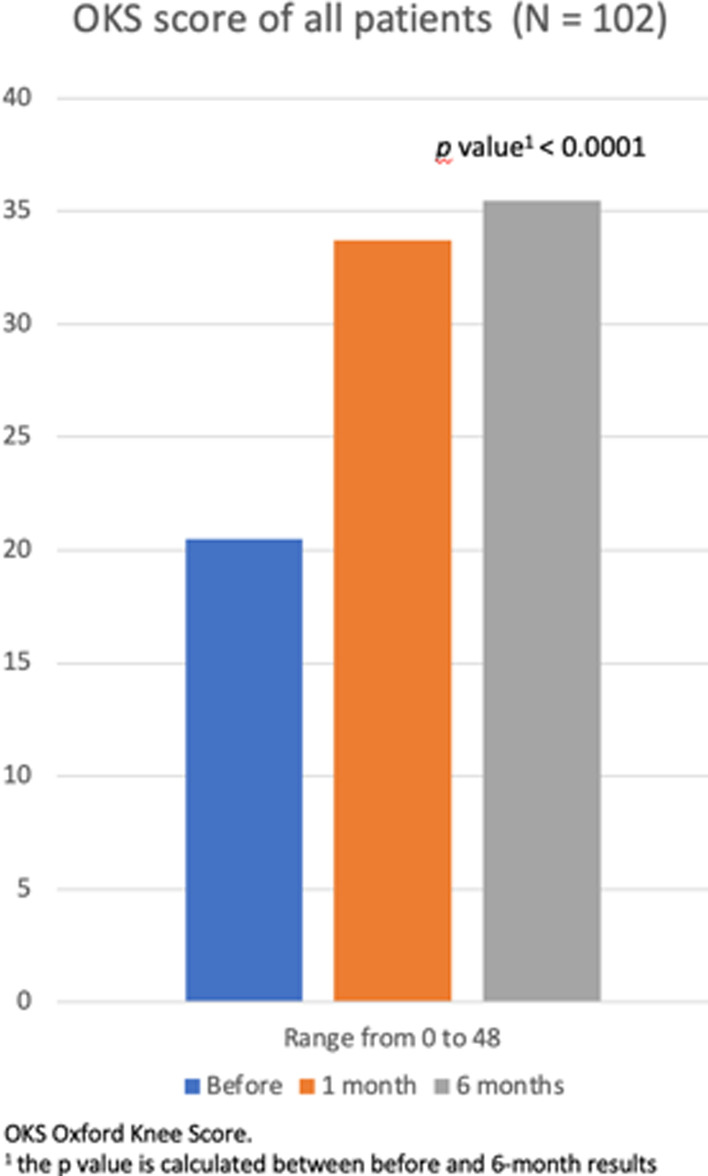
OKS

Knee OKS scores in the two treatment groups improved in similar ways (Fig. 15). The overall increase during follow-up in OKS scores of all patients was significant (*p* < 0.0001). The mean OKS score of all patients was 20.5 ± 5.2 points at pre-procedure and 35.46 ± 10.59 at last follow-up (*p* < 0.0001). The difference in Knee OKS scores between BMAC and ADCSs groups at last follow-up is not statistically significant (Table 3). Both treatment groups demonstrated significant improvement from pre- to post-procedure in OKS scores (*p* < 0.0001) (Table 4).

**Table 4:** K–L classification system

	K–L 2 (*N* = 50)	K–L 3 (*N* = 38)	K–L 4 (*N* = 14)
KOOS symptoms^a^			
Before	51.47 ± 19.11	39.73 ± 16.74	42.4 ± 28.46
1 month	77.31 ± 14.83	66.69 ± 15.11	65.26 ± 21.42
6 months	83.84 ± 18.45	65.62 ± 21.56	70.02 ± 31.41
*p* value^b^	< 0.0001	< 0.0001	< 0.0001
KOOS pain^a^			
Before	41.99 ± 17	35.12 ± 15.30	43.92 ± 24.92
1 month	75.6 ± 13.93	64.04 ± 14.90	66.36 ± 21.18
6 months	79.45 ± 18.61	64.48 ± 23.56	71.34 ± 30.46
*p* value^b^	< 0.0001	< 0.0001	< 0.0001
KOOS functional^a^			
Before	44.47 ± 18.68	38.35 ± 14.47	28.1 ± 15.98
1 month	77.81 ± 14.34	65.82 ± 16.34	71.12 ± 20.82
6 months	79.83 ± 19.96	64.3 ± 23.21	77.7 ± 28.52
*p* value^b^	< 0.0001	< 0.0001	< 0.0001
KOOS sport^a^			
Before	25.5 ± 22.12	17.78 ± 14.77	23 ± 25.40
1 month	61.5 ± 20.27	48.05 ± 19.03	51 ± 27.25
6 months	62.1 ± 27.65	44.41 ± 30.61	56 ± 40.06
*p* value^b^	< 0.0001	< 0.0001	< 0.0001
KOOS QoL^a^			
Before	27.60 ± 14.67	20.06 ± 14.58	17.7 ± 16.39
1 month	60.64 ± 18.72	50.59 ± 17.94	52.5 ± 28.50
6 months	66.76 ± 21.87	48.16 ± 25.49	60 ± 33.54
*p* value^b^	< 0.0001	< 0.0001	< 0.0001
VAS score^a^			
Before	6.7 ± 1.30	6.78 ± 1.44	6.2 ± 1.30
1 month	2.9 ± 1.16	3.05 ± 0.80	3.2 ± 1.30
6 months	2.53 ± 1.35	3.53 ± 2.18	3.4 ± 1.52
*p* value^b^	< 0.0001	< 0.0001	< 0.0001
OKS score^a^			
Before	20.68 ± 8.70	16.06 ± 5.26	14.2 ± 6.18
1 month	34.53 ± 6.36	29.72 ± 6.54	29.6 ± 5.94
6 months	37.05 ± 9.78	28.59 ± 9.56	27.6 ± 10.21
*p* value^b^	< 0.0001	< 0.0001	< 0.0001

*KOOS* Knee Injury and Osteoarthritis Outcome Score, *QoL* Quality of Life, *VAS* Visual Analogical Scale, *OKS* Oxford Knee Score

^a^Data was expressed by mean ± standard deviation

^b^*p* value is calculated between before and 6-month results

### Kellgren and Lawrence system

The mean knee KOOS score, knee OKS score, and VAS pain score was different according to the grade of KOA based on the K–L classification (Table 4). Patients with K–L grade 2 showed better functional and clinical outcomes than patients with K–L grades 3 and 4 at the last follow-up (*p* < 0.0001).

## Discussion

According to the main findings of the present study, intra-articular ADSCs or BMAC orthobiologic therapy improves clinical and functional scores in patients with symptomatic KOA at 6 months of follow-up with similar efficacies. Patients with mild KOA (K–L 2) treated with BMAC and ADSCs injections have better clinical and functional results than patients with moderate and severe KOA (K–L 3/4).

Intra-articular MSCs for KOA may improve pain and function for 12 or 24 months, with no evidence of improvements in cartilage status in KOA [35, 36, 41, 46]. Conversely, a recent meta-analysis of 13 RCTs showed that intra-articular MSC injection was not superior to placebo in pain relief and minimum clinically important functional improvement for patients with symptomatic KOA [47]. Recently, Aletto et al. analysed the short-term clinical and functional results of 126 patients with early KOA treated with adipose-derived stem cells only and showed a statistically significant improvement of KOOS and VAS [48].

In MRI evaluations of cartilage repair, current evidence showed controversial results, with no improvement in cartilage status [49–51]. An accurate pre- and post-operative planning with MRI imaging must be obtained to evaluate the improvement of cartilage status [52]. In studies without adjuvant surgery, there was no significant improvement of cartilage status after intra-articular injection of MSCs, with no difference in terms of the WORMS score than baseline. Only one study, which compared HTO and microfracture with and without MCSs injection, reported improved cartilage status in the MSC group based on MRI evaluation at 12 months [51]. Intra-articular injection of MSCs after concomitant surgery showed significantly higher MOCART score than HTO and microfracture without MSCs injection. Therefore, future studies of intra-articular injection of MSCs are necessary to accurately assess at MRI the efficacy of MSCs on cartilage status in KOA.

Recent systematic reviews and network meta-analyses compared MCSs with other injectable intra-articular orthobiologic therapy [53, 54]. By 12 months, AD-MSCs and LP-PRP showed similar clinical pain relief effects, with better functional improvement with LP-PRP. Clinical efficacy of the hyaluronic acid viscosupplementation was lower than that of biological agents.

The total number of MSCs present in bone marrow harvests and lipoaspirates has not yet been estimated [55]. MSCs collected from bone marrow aspirate form only a small percentage of mononuclear cells, approximately 0.001–0.02% [56]. Also, age could represent a limit for autologous harvest, in terms of the fitness of aspirate stem cell concentrate. After the age of 75, the proliferative capacity of mesenchymal cells is reduced [57]. Thus, the clinical effect of BMAC and ADSCs is most likely exerted through some concentration of MSCs in combination with angiogenic, anti-inflammatory, and immune-modulatory cytokines and growth factors [58]. From a surgical point of view, bone marrow harvesting and lipoaspiration are both simple procedures with minimal side effects. Both procedures are minimally invasive, last about 30 min, and can be carried out as an office procedure.

This study has some limitations. First, the choice of treatment was not randomized. Furthermore, there was no comparison with other therapies, such as placebo or other injection such as hyaluronic acid, PRP, and corticosteroid. Another important limitation of this study was the short- term follow-up. Also, we evaluated only patient-reported outcome measures, without considering the biological effects of mesenchymal cells on cartilage repair, which can be assessed by MRI. Only another study directly compared the results between autologous BMAC or ADSCs as tissue sources of MSCs for symptomatic KOA and showed significant improvement in clinical outcomes with both BMAC and ADSCs injections, without a significant difference between the two autologous tissue sources [42]. We are aware that the highest level of evidence for effectiveness of one or the other treatment outlined in the present investigation can only be produced using a randomized study trial design. However, given the constraints of our setting, we are confident that the results are valid and reliable. The recruitment process was rigorous, data collection was performed in a strict scientific fashion, we used validated outcome measures, and the results obtained are clinically relevant. Future randomised clinical trials with longer follow-up should investigate which autologous orthobiologic tissue source is most effective in KOA.

## Conclusions

Both BMAC and ADSC intra-articular injections significantly improved pain and functional outcomes at 6-month follow-up in patients with KOA. There were no statistically significant differences between BMAC and ADCSs groups in terms of clinical and functional outcomes. Further high-quality clinical trials are required to validate these results on a larger scale.

## Data Availability

The datasets generated during and/or analysed during the current study are available as reasonable request to Mr. Pintore (apintore@unisa.it).
